# Time distortion under threat: Sympathetic arousal predicts time distortion only in the context of negative, highly arousing stimuli

**DOI:** 10.1371/journal.pone.0216704

**Published:** 2019-05-13

**Authors:** Ruth Sarah Ogden, Jessica Henderson, Francis McGlone, Michael Richter

**Affiliations:** School of Natural Sciences, Liverpool John Moores University, Liverpool, United Kingdom; Boston Children's Hospital / Harvard Medical School, UNITED STATES

## Abstract

Theoretical models of time perception suggest a simple bottom-up relationship between physiological arousal and perceived duration. Increases in physiological arousal lengthen the perceived duration of events whereas decreases in physiological arousal reduce them. Whilst this relationship has been demonstrated for highly arousing negatively valenced stimuli, it has not been demonstrated for other classes of distorting stimuli (e.g. positively valenced or low arousal stimuli). The current study tested the effect of valence (positive and negative) and arousal level (high and low) on the relationship between physiological arousal and perceived duration. Sympathetic nervous system (SNS) and parasympathetic nervous system (PSNS) activity was measured during a verbal estimation task in which participants judged the duration of high and low arousal, positive, negative and neutrally valenced IAPS images. SNS and PSNS activity were indexed by measuring Pre-Ejection Period (PEP) and High Frequency Heart-rate Variability (HF-HRV) respectively. SNS reactivity was predicative of perceived duration, but only for high arousal negatively valenced stimuli, with decreases in PEP being associated with longer duration estimates. SNS and PSNS activity was not predictive of perceived duration for the low arousal negative stimuli or the low and high arousal positive stimuli. We therefore propose a new model suggesting that emotional distortions to time result from a combination of bottom-up (physiological arousal) and top-down (threat detection) factors.

## Introduction

The ability to accurately judge the duration of events in the milliseconds to seconds range has been suggested to be critical to behavioural regulation and survival [1, 2, 3]. Indeed experimental work shows that the timing of behaviours performed in the milliseconds-seconds range, for example eye-contact duration [4] and pauses in speech [5], influence the interpretation and success of these social acts. Millisecond perceptual accuracy has also been shown to be important when making experimental [6] and applied [7] judgments of time to contact and collision. Despite this, our perception of duration is distorted by affective state (emotion) resulting in the subjective shortening and lengthening of the perceived duration of emotional events [2, 8].

The most consistently reported emotional distortion to time is that of negatively valenced stimuli which, when experienced, are perceived as lasting for longer than neutral stimuli of the same duration. The effect is evident across modalities, being observed in studies using negatively valenced static images of faces [9], complex scenes [10], taboo words [11] dynamic visual stimuli [12], negatively valenced auditory stimuli [3] and negatively valenced somatosensory stimuli (i.e. pain) [13, 14]. It is also observed in clinical groups, for example, people with phobia over-estimate the duration of phobia triggers relative to non-phobic’s [15, 16]. Whilst negative valence is associated with a lengthening of perceived duration, positive valence has been associated with a shortening of perceived duration. For example, the duration of positive images [17] and happy music [18] are perceived as shorter than neutral stimuli presented for the same amount of time. Similarly, the experience of pleasant affective touch shortens the perceived duration of concurrent events [19].

Emotional distortions to time have traditionally been explained through changes in physiological arousal. Popular models of temporal perception, for example Scalar Expectancy Theory (SET) [20] and the Striatal Beat Frequency Model (SBF) [21] propose a direct bottom-up relationship between physiological arousal and perceived duration. Increases in physiological arousal lengthen the perceived duration of events and decreases in physiological arousal shorten the perceived duration of events [2, 8]. In SET, arousal acts directly on the pacemaker which forms the raw representation of duration [22]. Increases in arousal are thought to increase pacemaker output rate, leading to longer perceptions of duration whereas decreases in arousal are thought to reduce pacemaker output rate, leading to shorter perceptions of duration. In SBF, emotional distortions to time are thought to occur due changes in dopamine [23]. Emotional stimuli induce changes in phasic dopamine levels in the cortex, prefrontal cortex and striatum [24]. These changes are thought to alter the firing rate of cortical projections to the striatum, resulting in changes in the rate of cortical oscillations and distortions to time [2]. Increases in dopamine increase firing and oscillation rate, resulting in longer perceptions of duration whereas decreases in dopamine slow the firing and oscillation rate leading to shorter perceptions of duration.

In Craig’s Homeostatic Model of Timing, changes in physiological arousal are hypothesised to affect timing because of the dual role of the anterior insular cortex (AIC) in timing-related aspects of self-representation and homeostasis of the autonomic nervous system (ANS) [25–27]. During homeostatic regulation, the AIC is asymmetrically activated; right-side activation is associated with increased activity of the sympathetic branch (SNS) of the ANS whereas the left-side is associated with reduced SNS activity and increased activity of the parasympathetic branch (PSNS) of the ANS. The AIC is also activated during temporal perception [28] leading to the suggestion that it enables the body and self to be represented “in time” [25]. Distortions to time occur when the AIC performs temporal and homeostatic processing simultaneously [26]. In circumstances in which SNS activity increases, the associated increased right-side AIC activity leads to a subjective lengthening of time. However, in states where there is a lack of SNS activity and greater PSNS activity, the increased left-side AIC results in a perceived shortening of time.

These three models all imply a bottom-up effect of arousal on time perception in which the physiological consequences of encountering highly arousing negatively valenced stimuli (i.e. an increase in physiological arousal) affect the subsequent processing of duration. Critically, the bottom-up relationship between arousal and perceived duration proposed in these models implies that *any* increase in physiological arousal should subjectively lengthen perceived duration and *any* reduction in physiological arousal should subjectively shorten perceived duration. However, recent studies directly testing the relationship between physiological arousal (SNS and PSNS reactivity) and emotional distortions to time, have failed to demonstrate this universally for all types of emotional stimuli [29, 30].

van Hedger et al. (2017) [29] tested the relationship between SNS (indexed by Pre-ejection period) and PSNS activity (indexed by HF-HRV) and distortions to perceived duration by examining the effect of a social stressor on temporal reproductions of neutral, negative and positive IAPS images. Participants reproduced the duration of positive negative and neutral IAPS images presented for 400, 1,650, 2,900, or 4,150 ms before and after performing a social stressor test in which they had to prepare and deliver a speech to camera. The findings showed that, for the shortest stimulus duration (400ms), there was a significant correlation between changes in reproduction durations for the negative images (before and after the stressor) and changes in SNS activity. No relationships were found between SNS activity and reproductions of longer negative stimuli or any of the positive or neutral stimuli. There were also no relationships between PSNS and any reproduction. Although these findings, at first glance, indicate that the relationship between SNS activity and perceived duration is unique to negatively valenced stimuli, a number of methodological issues prevent this conclusion. Firstly, PSNS and SNS activity were not recorded separately for the positive, negative and neutral stimuli. It is therefore impossible to determine whether reaction to a specific class of stimuli was related to temporal distortions for that class of stimuli. Secondly, the use of a reproduction method, which prevented a state change occurring between learning the duration and testing the duration [31], likely limited the emergence of any distortion to time. Further research establishing the precise relationship between the physiological response to a class of emotional stimuli, and the distortion to time that they produce is therefore required.

More recently, Piovesan et al., (2018) [30] tested the relationship between SNS (indexed by skin conductance level) and PSNS activity (indexed by HF-HRV) and distortions to the perceived duration of painful stimuli. In two experiments, participants estimated the duration electro-cutaneous stimulation delivered at levels inducing no pain, low pain and high pain (experiment 1), or, participants estimated the duration of a neutral visual stimulus whilst experiencing continuous thermal pain delivered at levels inducing no pain, low pain and high pain (experiment 2). All to-be-timed-stimuli were presented for between 200ms and 1300ms. The results showed that when pain was itself the to-be-timed-stimulus, and therefore deemed task relevant, perceived duration was predicted by SNS reactivity but not PSNS activity. However, when pain was no itself the to-be-timed stimulus, and therefore deemed task irrelevant, neither SNS or PSNS activity were predictive of perceived duration.

Taken together, the findings of van Hedger et al., (2017) [29] and Piovesan et al., (2018) [30] suggest that there is not a clear evidence for a universal relationship between SNS-PSNS activity and perceived duration. Instead, there is emerging evidence to suggest that factors such as stimulus valence and task relevance determine whether SNS activity is directly related to duration distortion. This implies that a simple bottom-up arousal model of emotional distortions to time may be inaccurate or overly simplistic.

A further issue for a simple bottom-up arousal model is its capacity to explain the subjective shortening of the perceived duration of positively valence stimuli [17–19]. Physiological and neuroimaging studies show that positively valenced stimuli can increase SNS reactivity in a manner comparable to the increases observed for negatively valenced stimuli, particularly when the stimuli are highly arousing (e.g. erotic images and mutilated bodies) [32]. A simple bottom-up arousal model would therefore predict that the perceived duration of positive stimuli should be lengthened because SNS activity has increased, rather than shortened as is typically observed in experiments.

Collectively, these findings suggest that the relationship between physiological arousal and temporal distortions is more complex than previously suggested. Whilst a simple direct relationship between physiological arousal and perceived duration is evident for highly arousing negatively valence stimuli [29, 30], such a relationship is absent for positively valenced stimuli [29] and task-irrelevant sources of arousal [30]. A universal bottom-up cause to emotional distortions to time is therefore incompatible with experimental observations.

An alternative model is that emotional distortions to time result from a combination of bottom-up (physiological arousal) and top-down (cognitive evaluation of the stimulus) processes. Here, top-down processes reflect the evaluation and appraisal of the semantic content or meaning of to-be-timed-stimulus based on stored knowledge. This may include interpreting the valence of the stimuli (positive or negative effect), the emotion being expressed or the presence or absence of threat. Contemporary theories of emotion acknowledge that *both* bottom-up and top-down processes are involved in emotion experience [33, 34]. Indeed, neuroimaging studies suggest distinct but overlapping neural networks for the top-down and bottom-up elements of emotion experience [33]. It therefore seems plausible that both top-down and bottom-up mechanisms would have the potential to influence emotional distortions to time.

In emotional distortions to time, top-down processing may determine whether changes in arousal related SNS-PSNS activity influence the perceived duration of emotional events [30]. This type of model is proposed in Piovesan et al., (20180) [30] to explain the absence of a relationship between task-irrelevant changes in SNS-PSNS activity and perceived duration in Experiment 2 of their paper. Piovesan et al., (2018) [30] suggested that for emotional distortions to time to maintain their perceptual and cognitive advantages for survival (2, 3, 26) the effect of physiological arousal on perceived duration must be limited to times of threat. This is because it would not be adaptive if irrelevant, non-threatening changes in physiological arousal caused time to wax and wane during day-to-day activity. A top-down process is therefore required to identify these situations and to prevent SNS-PSNS activity influencing time in the absence of threat. Lake et al., (2016) [2] make similar suggestions when discussing “biological relevance”, suggesting that differing effects of different emotional states on time perception may be due to the biological relevance of each individual state, with high arousal threat stimuli possessing more biological relevance than other classes of stimuli. Biological relevance therefore acts as a mechanism to explain why different classes of equally arousing emotional events can have different effects on perceive duration.

An evolutionary based model in which temporal distortions are determined by top-down and bottom-up processes enables us to make specific predictions about when a direct relationship between SNS-PSNS activity and perceived duration should be observed. Only one of the four possible combinations of valence and arousal does actually represent a threat to survival: a negatively valenced stimuli that induces a lot of arousal. In this situation, perceived duration should be predicted by physiological reactivity to the stimulus (i.e. increased SNS and reduced PSNS response). However, when stimulus valence is negative but arousal is low perceived duration should *not* be predicted by physiological reactivity to the stimulus because there is no immediate threat to survival. Similarly, because neither high nor low arousal positive stimuli present a threat to survival the perceived duration of these stimuli should not be predicted by the SNS-PSNS arousal that they evoke.

The findings of van Hedger et al., (2017) [29] and Piovesan et al., (2018) [30] provide some support for these suggestions as both studies only observed that SNS-PSNS activity was predictive of the perceived duration of high arousal negative stimuli. However, because each study manipulated either valence *or* arousal, but not both together, a comprehensive assessment of the effect of stimulus valence and stimulus arousal level is currently absent. For example, Piovesan et al., (2018) [30] manipulated arousal by exposing participants to low and high levels of pain but did not present any positively valenced stimuli. Conversely, van Hedger et al. (2017) [29] manipulated valence but did not control for arousal. Furthermore, because van Hedger et al., did not record physiological arousal separately for each stimulus valence, a direct test of the relationship between physiological arousal uniquely evoked by positively valenced stimuli and distortions to the duration of positive stimuli is lacking. To comprehensively test the predictions of the bottom-up top-down model of emotional distortions to time a systematic evaluation of how stimulus valence and arousal affect the relationship between arousal and temporal distortions is required.

### The current study

The current study tested whether stimulus valence (positive or negative) and stimulus arousal level (high and low) affected the relationship between physiological arousal (SNS and PSNS reactivity) and perceived duration. Participants were required to estimate, in milliseconds, the duration of five classes of images taken from the International Affective Pictures System (IAPS) [35]: low arousal positive, low arousal negative, high arousal positive, high arousal negative and neutral. Images were delivered in blocks of the same stimulus class (i.e. a block of high arousal negative, a block of high arousal positive) and recordings of sympathetic and parasympathetic activity were taken throughout each block separately. Sympathetic arousal was indexed by pre-ejection period (PEP) [36]. Parasympathetic arousal was indexed by high frequency heart rate variability (HF-HRV) [37, 38]. For both PEP and HF-HRV, the change from baseline was then calculated for each block. This block based design replicates the experimental designs of van Hedger et al., (2017) [29] and Piovesan et al., (2018) [30]. The relationship between temporal estimates, SNS and PSNS reactivity was then tested. This type of individual differences approach to understanding emotional distortions to time, in which objective measures of arousal are taken (i.e. SNS-PSNS activity) and stimulus arousal *and* valence are manipulated, was recently advocated by Lake et al., (2016) [2] as the preferred strategy for establishing the underlying causes of emotional distortions to time.

If the existing simple bottom-up arousal model of emotional distortions to time is correct then significant relationships between physiological measures (SNS or PSNS activity) and perceived duration should be observed in the high and low arousal, positive and negative conditions. However, if the top-down bottom-up model of emotional distortions to time is correct then significant relationships between physiological measures (SNS or PSNS activity) and perceived duration should only be observed for the high arousal negative condition in which threat is present. In the low arousal negative conditions and high and low arousal positive conditions there should be no relationship between SNS-PSNS activity and perceived duration because of the absence of immediate threat.

## Method

### Participants

Fifty participants, with no known cardiac pathology, were recruited through volunteer sampling from Liverpool John Moores University and the general population. Sample size for this study was similar to that used in previous research examining the relationship between physiological arousal and distortions to the perception of time [29, 30] and a Cardiac pathology was assessed via a questionnaire. Participants who indicated a known cardiac pathology were not invited for further participation. Participants were recruited from the undergraduate and post-graduate community of Liverpool John Moores University via email advertisement. Participants were given a £10 shopping voucher in exchange for participation. Participants were aged 18 to 35 (M = 20.68, SD = 3.37) with 37 females and 13 Males. All participants gave informed consent. The study was approved by Liverpool John Moores University Research Ethics Committee and all participants gave informed written consent. The study was conducted in accordance with the principles expressed in the Declaration of Helsinki.

### Materials and apparatus

*Physiological recording*: A medis Cardioscreen 1000 sampled ECG and ICG signals at a sampling rate of 1000 Hz to determine pre-ejection period (PEP) and high frequency heart rate variability (HF-HRV). The Cardioscreen 1000 impedance cardiograph electrodes were connected to the base of the participant’s neck and to the middle axillary lines at the level of the xiphoid on both the right and left sides, the earlobe electrode was connected to the left earlobe. SNS arousal was indexed by PEP. PEP refers to the period between the electrical innervation of the ventricular myocardium (Q wave of the electrocardiogram [ECG]) and the opening of the aortic valve (B notch), and is the best, non-invasive indicator of SNS impact on the heart that is available [36, 37]. PSNS arousal was indexed by HF-HRV. HF-HRV reflects respiratory sinus arrhythmia (the increase and decrease of heart rate during inhalation and exhalation, respectively), which is directly determined by the PSNS [37]. A V100 blood pressure monitor assessed participants systolic and diastolic blood pressure to control for afterload effects on PEP [36].

*Stimulus selection*: Stimuli were selected from the IAPS [35]. Five categories of images were selected which constituted the five experimental conditions: high arousal negative, high arousal positive, low arousal negative, low arousal positive, and neutral. Six images selected for each condition (see Table 1 for image numbers). The images were selected according to IAPS standard ratings for arousal and for valence. High arousal negative images (valence 1.50–2.50, arousal 6.00–7.50), high arousal positive images (valence 6.50–8.00, arousal 6.00–7.50), low arousal negative (valence 1.75–3.25, arousal 3.50–4.75), low arousal positive (valence 7.00–8.50, arousal 3.25–4.75) and neutral (valence 4.00–5.00, arousal 1.50–3.00). These criteria were informed by that used in Angrilli et al., (1997) [38] and Gil & Droit-Volet, (2012) [10]. When multiple images met the criteria for selection, images used in previous studies [10] and [39] were preferentially selected. For the high arousal negative condition, images consisted of mutilated human and animal bodies which have been previously shown to evoked freeze responses [40] and startle responses [41] consistent with threat. Table 2 shows the mean valence and arousal scores for each condition taken from the IAPS manual [35].

**Table 1:** IAPS numbers.

*High Arousal Negative*	*High Arousal Positive*	*Low Arousal Negative*	*Low Arousal Positive*	*Neutral*
3110	4660	9290	1750	7010
3120	4680	9330	2050	7050
9050	4690	9001	1600	7060
9405	4668	9832	1610	7150
9410	8080	2750	5551	7175
9921	8200	2205	1460	7031

**Table 2:** Valence and arousal means and standard deviations for image levels.

*Image type*	*Valence M*	*Valence SD*	*Arousal M*	*Arousal SD*
High Arousal Negative	1.69	0.27	6.76	0.47
High Arousal Positive	7.24	0.41	6.47	0.42
Low Arousal Negative	2.67	0.53	4.27	0.35
Low Arousal Positive	7.73	0.34	3.83	0.52
Neutral	4.75	0.18	2.22	0.47

*Verbal estimation task*: All stimuli were presented on a 32 inch monitor. Participants used the keyboard to enter all responses. The experiment structure was written in E-Prime 2.0 which controlled all experimental events.

### Procedure

The basic experimental procedure was as follows; cardiac monitors were attached to the participant. Participants were then informed that they would complete the following two tasks, five times, once for each condition (high arousal positive, high arousal negative, low arousal positive, low arousal negative, neutral): 1) a five minute baseline recording of cardiac activity and 2) a verbal estimation task. The order of the conditions was randomised across participants.

*Baseline cardiac recording*: Each verbal estimation task was preceded by a 5 minute baseline recording period in which participants listened to one of the following audiobooks, Wonderful Wizard of Oz, Peter Pan, The Jungle Book, or Alice in Wonderland. Physiological recordings were taken throughout. During baseline recordings participants were asked to sit in a comfortable position and listen to the story.

*Verbal estimation task*: The verbal estimation paradigm was based on that used in previous research examining the effects of emotion [10, 14, 19, 30] and pre-stimulus stimulation [22, 42] on time perception. Participants were informed that they would be presented with a series of images on the screen and that their task was to judge how long each image lasted for. Before starting the task participants were informed that all stimuli would be between 50 and 1000ms. Participants were reminded that 1000ms equals one second, 500ms therefore equals half a second and 250ms equals a quarter of a second. At the beginning of each trial participants were instructed to press the spacebar to begin. An inter stimulus interval (ISI) of either 1000 or 1500ms was then interposed. One of six IAPS images was then presented, the image presented was selected at random by E-Prime. The image was presented for one of seven standard durations: 200, 300, 400, 500, 600, 700, or 800ms. A 500ms ISI was then interposed following which, participants were instructed to type their estimate of the images presentation duration in milliseconds. An onscreen message reminding participants that their estimate should be between 50 and 1000ms and that 1000ms equalled one second, was displayed whilst the estimates were made. Following estimation and ISI of 1000ms was interposed. Participants completed 49 trials in each verbal estimation task. Trial order was randomised for each participant. No performance feedback was given. Each block of the verbal estimation task took approximately six minutes to complete. A break of 5 minutes was interposed between each block, before the next period of baseline recording. Physiological recordings were taken throughout. The whole experiment lasted for approximately 1 hour 30 minutes.

### Data analysis

#### Physiological measures

Our analysis of PEP and HF-HRV followed the procedure described in Richter (2010) [43]. R-peaks were detected in the ECG signal using a modified Pan-Tomkins peak detection algorithm. The ICG signal was differentiated and the resulting dZ/dt signal was filtered with a low-pass Butterworth filter with a corner frequency of 50 Hz. The filtered dZ/dt signal and the detected r-peaks were used to create dZ/dt ensemble averages over intervals of 1 minute [44]. PEP was scored as the interval between R-onset and the B-point following the guidelines of the Society for Psychophysioloigcal Research [37]. Drawing on the detected r-peaks, IBIs were extracted, resampled at 4Hz, detrended using smoothness priors [45], and the high frequency (0.15 Hz– 0.40 Hz) power spectrum was determined using Fast-Fourier Transformation with Welch’s periodogram method (256 samples window width, 50% window overlap) [37, 38]. This analysis was conducted using Bluebox [43] and Kubios HRV Analysis software. These calculations were conducted on the baseline data and the data from the five conditions of the verbal estimation tasks. All data was then baseline corrected by subtracting the PEP and HF-HRV scores recorded during the baseline of each condition from that recorded during the verbal estimation task of the associate condition. This gave five baseline corrected scores, one for each of the five classes of stimuli (neutral, high and low, positive and negative images).

To determine the relative effect of the high and low arousal, positive and negative stimuli on SNS and PSNS activity, we calculated change scores reflecting the difference in (baseline corrected) SNS and PSNS activity between the emotion inducing conditions (high arousal negative, low arousal negative, high arousal positive, low arousal positive) and the non-emotional neutral condition. In particular, change scores were calculated by subtracting the baseline correct PEP and HF-HRV scores of the neutral stimuli condition from the baseline corrected PEP and HF-HRV scores of the emotional stimuli conditions (high and low, positive and negative). So, for example, the change score for the high arousal negative stimuli was determined by deducting the average baseline corrected PEP and HF-HRV during completion of the neutral stimuli verbal estimation task from the average baseline corrected PEP and HF-HRV during completion of the high arousal negative stimuli verbal estimation task. A change score of zero therefore indicates that the PEP or HF-HRV response to the emotional stimuli was of the same magnitude than the response to neutral stimuli. A negative PEP change score reflects a stronger decrease in PEP (increase in SNS activity) in the emotional stimuli condition in comparison with the neutral stimuli condition whereas a positive change score indicates a stronger increase in PEP compared to the neutral condition. For HF-HRV, a negative change score indicates a stronger decrease in HF-HRV (decrease in PSNS activity) in the emotional stimuli condition (compared to the neutral stimuli condition) whereas a positive change score indicates a stronger increase in HF-HRV.

#### Verbal estimation data

For the duration data, mean verbal estimates were calculated for each stimulus in each condition. These were then summed to produce a total estimate score for each condition (see 30). To calculate the effect of stimulus condition on time estimate, the difference in estimates from the neutral condition to the high positive, low positive, high negative, low negative was calculated, by deducting the total estimate for the neutral condition from the total estimate for the emotional condition to give measures of “duration change”. A change score with a value of zero therefore indicates no difference in estimates between the neutral and the emotional condition. A positive value indicates a longer estimate for the emotional condition than the neutral stimuli and a negative value indicates a shorter estimate for the emotional condition than the neutral condition.

## Results

### The relationship between SNS and PSNS activity and emotional distortions to time

Table 3 shows Pearson’s correlation coefficients of the relationship between change scores for time estimation, PEP and HF-HRV. There was a significant negative relationship between PEP change and estimate change for the high arousal negative condition. Estimate change for the low arousal negative and high and low arousal positive conditions were not related to PEP change. The correlation obtained between PEP change and estimate change was significantly greater in the high arousal negative condition than in the low arousal negative condition (*p* = .03), the high arousal positive condition (*p* = .03) and the low arousal positive condition (*p* = .03). There were no significant relationships between HF-HRV change and time estimation change in any of the conditions.

**Table 3:** Correlation coefficients between time estimate change, PEP change and HF-HRV change.

*Valence*	*Arousal*	*PEP*	*HF-HRV*
Negative	High	-.40*	-.13
	Low	-.04	.15
Positive	High	-.05	.15
	Low	-.05	-.02

* = *p* = .004

Multiple regression tested whether changes in PEP and HF HRV predicted changes in duration estimates. For the high arousal negative condition, ANS reactivity explained 12.90% of the variance in the increase in duration estimates from the neutral condition (*R*^2^ = 16.50, *F*(2, 49) = 4.63, *p* = .02). PEP was a significant predictor (β = -.39, *p* = .006) but HF HRV was not (β = -.06, *p* = .69). No significant model fits were found for the low arousal negative condition *F*(2, 49) = .50 *p* = .61, the high arousal positive condition *F*(2, 49) = .62 *p* = .54, or the low arousal positive condition *F*(2, 49) = .09, *p* = .91.

### The effect of stimulus on estimates, PEP and HF-HRV

To establish whether the high and low arousal positive and negative stimuli distorted time and induced changes in physiological arousal compared to neutral stimuli, one sample t-tests were conducted on time estimates, PEP and HF-HRV change scores in each one of the four high and low arousal negative and positive conditions. These scores are shown in Table 4.

**Table 4:** Descriptive statistics of the change from neutral for time estimates, PEP and HF-HRV for the four conditions.

*Valence*	*Arousal*	*Time Change (SD)*	*PEP Change (SD)*	*HF-HRV Change (SD)*
Negative	High	130.10 (411.67)	-1.02 (3.57)	1.89 (19.01)
	Low	129.38 (449.14)	-1.23 (4.32)	-1.59 (18.49)
Positive	High	63.18 (566.34)	-1.85 (4.54)	3.20 (23.34)
	Low	27.48 (543.06)	-1.11 (6.31)	-.62 (17.99)

Change scores for time estimates differed significantly from zero in the high arousal negative *t*(49) = 2.24, *p* = .03 and low arousal negative conditions *t*(49) = 2.04, *p* < .05 indicating that the perceived duration of the low and high arousal negative images was subjectively longer than that of the neutral stimuli. Change scores did not differ from zero in the high arousal positive *t*(49) = .79, *p* = .43 and low arousal positive conditions *t*(49) = 36, *p* = .72 indicating that the perceived duration of the positive stimuli was not distorted relative to neutral.

Change scores for PEP differed significantly from zero in the high arousal negative *t*(49) = -2.03, *p* = .05, low arousal negative *t*(49) = -2.01, *p* < .05 and high arousal positive conditions *t*(49) = -2.89, *p* = .006. In all instances, PEP values were significantly lower than zero indicating that SNS reactivity increased in high arousal positive and negative conditions and the low arousal negative condition relative to neutral. Change scores did not differ from zero in the low arousal positive condition *t*(49) = -1.25, *p* = .22 indicating that SNS reactivity was comparable in the low arousal positive and neutral conditions.

Change scores for HF-HRV did not differ from zero in the high arousal negative *t*(49) = .70, *p* = .49, low arousal negative *t*(49) = -.61, *p* = .55, high arousal positive *t*(49) = .97, *p* = .34 and low arousal positive *t*(49) = -.24 *p* = .81. PSNS activity was therefore comparable in all conditions.

## Discussion

This study tested the effect of stimulus valence (positive and negative) and stimulus arousal level (high and low) on the relationship between physiological arousal and distortions to perceived time. Physiological arousal was defined as SNS reactivity (indexed by PEP) and PSNS reactivity (indexed by HF-HRV). Distortion to time was defined as the difference between estimates given for the neutral stimuli and the high and low, positive and negative stimuli.

The results show that the perceived duration of the high and low arousal negatively valenced stimuli was significantly longer than the perceived duration of the neutral stimuli. This replicates previous findings that the duration of negatively valenced stimuli are overestimated relative to neutral stimuli [2, 8]. PEP scores were also significantly lower in the high and low arousal negative conditions than the neutral condition, confirming that both conditions increased SNS reactivity. HF-HRV did not change from the neutral to the high or low arousal negative conditions, suggesting that neither stimuli affected PSNS activity. For the high arousal negative condition, there was a significant relationship between duration change and PEP change suggesting that increases in SNS activity were associated with longer subjective perceptions of duration for the high arousal negatively valenced stimuli. There was no relationship between HF-HRV and duration change, suggesting that PSNS reactivity was unrelated to the perceived duration of the stimuli. These findings replicate those reported in Piovesan et al., (2018) [30] using highly and moderately painful electro-cutaneous stimuli. Interestingly, although the low arousal negative stimuli significantly increased SNS reactivity and lengthened duration estimates, no relationships were observed between duration change and SNS or PSNS reactivity. The change in physiological arousal evoked by the low arousal negative stimuli was not therefore predictive of the distortion to its perceived duration. This finding is incompatible with the simple bottom-up model of emotional distortions to time which would have predicted a significant relationship.

Duration estimates for the high and low arousal positive stimuli did not differ from the estimates for the neutral stimuli. Therefore, regardless of stimulus arousal level, we failed to replicate previous observations of distortions to the perceived duration of positive stimuli [17–19, 39, 45]. Although the majority of previous studies have observed a relative shortening of the perceived duration of positive stimuli this effect is not universally observed. For example Droit-Volet et al., (2004) [9] did not observe underestimation of duration for happy facial images compared with neutral ones and Angrilli et al., (1997) [39] reported that high arousal positive stimuli were perceived as lasting for longer than low arousal negative stimuli. Future systematic evaluation of how positive affect influences duration perception is therefore warranted.

Interestingly, despite observing no effect of positive valence on duration perception in the high arousal positive condition, PEP differed significantly from the neutral condition, indicating an increase in SNS activity. This confirms previous suggestions that high arousal positive stimuli increase SNS reactivity [32]. HF-HRV did not differ from the neutral condition indicating that PSNS reactivity was unaffected by the high arousal positive condition. Furthermore, there was no relationship between SNS or PSNS activity and perceived duration. For the low arousal positive condition, however, there was no significant change from neutral for the duration estimates, SNS activity or PSNS activity. There was also no significant relationship between SNS or PSNS activity and perceived duration. Therefore, for positively valenced stimuli regardless of arousal levels, we failed to establish any relationships between physiological reactivity and the perceived duration of positive stimuli. These findings therefore replicate van Hedger et al’s (2017) [29] observation that the perceived duration of positive stimuli are not related to SNS or PSNS activity. These findings are incompatible with the bottom-up theory of emotional distortions to time which would have predicted that the increase in SNS activity resulting from the high arousal positive stimuli should have resulted in a subjective lengthening of perceived duration.

Collectively, the differing relationships between physiological arousal and distortions to time observed for the high and low arousal, positive and negative conditions shows, for the first time, the differential effects of arousal and valence on perceived duration. When arousal is low, regardless of stimulus valence, SNS and PSNS activity are not predictive of duration distortion. When arousal is high however, stimulus valence determines whether physiological arousal is predictive of perceived duration. Indeed, SNS reactivity is only predictive of distortions of the perceived duration of highly arousing negatively valenced events and not positively valenced ones. Together these findings highlight the important of conducting experiments that manipulate arousal *and* valence to better understand the causes of distortions to duration.

Comparison of the results from the high and low arousal, positive and negative conditions of this study suggest that the simple bottom-up arousal model of emotional distortion to time is incompatible with the data. In the low arousal negative condition, both SNS reactivity and perceived duration were greater than in the neural condition, but the two were unrelated. In the high arousal positive condition, SNS reactivity was greater than in the neutral condition, but no distortion to time was observed. It would therefore seem that there is not a universal relationship between physiological and distortions to the duration of emotional events.

The findings are however compatible with the predictions of the top-down and bottom-up model of emotional distortions to time. As hypothesised, SNS-PSNS activity was only predictive of distortions to the perceived duration of high arousal negatively valenced stimuli. Previous research suggests that the types of images used in this study (i.e. mutilated bodies and images of dead people) reliably evoke freeze responses [40] and startle responses [41] consistent with suggestions that they induce threat avoidance and promote survival responding. When stimuli were non-threatening and lacking in biological relevance i.e. negatively valenced but low arousal or positively valenced, SNS-PSNS activity was not predictive of perceived duration. These findings confirm that changes in physiological arousal do not universally distort time. Instead, the effect of physiological arousal appears to be limited to circumstances of high biological relevance and threat, supporting suggestions that top-down stimulus evaluation (for threat) moderates the effects of physiological arousal on temporal distortions. This suggestion is supported on an anatomical level: the AIC, which has been suggested as a neural basis for temporal distortions [26], is specifically involved in the integration of bottom-up sensory/interoceptive information with top-down goal directed objectives [46]. We therefore speculate that AIC activation may determine which types of stimuli distort time.

If physiological arousal is not related to distortions to the duration of positive stimuli and low arousal negative stimuli, other factors must be causal in evoking emotional duration distortion in these circumstances. Emotional distortions to time are often explain by two forces; “arousal effects” or “attention effects” [8] which are often conceived of as acting independently of one another [2]. If arousal is not causal in some emotional distortions to duration it is possible that attentional effects produce distortions in these instances. Veridical timing requires adequate attention to duration, when inadequate attention is paid to time, for example, when distracted, perceived duration shortens [47]. Attentional affects are therefore typically used as explanations for the underestimation of the duration. For example, Ogden et al., (2014) [19] suggest that positive touch detracted attention away from timing, resulting in shorter perceptions of duration and Gil et al., (2009) [48] suggested that liked and disliked food images detracted attention away from timing, resulting in shorter perceived durations. In the current study however, because no stimuli were perceived as subjectively shorter than the neutral stimuli, it is difficult to apply this basic attentional account to the data.

Although attention and arousal are often treated separately within the field of temporal perception, there are well-established interrelations between the two: attention allocation can modulate arousal responses and arousal levels can moderate attention allocation and sustainability [49, 50]. Critically, heightened arousal can improve sustained attention on task relevant negatively valenced items [51]. It is therefore possible that, in the low arousal negative valance condition of the current study, the increase in SNS reactivity was not itself related to perceived duration, but instead improved attention across the task (relative to the neutral condition) leading to a lengthening of perceived duration. Further research should systematically examine how arousal and valence influence attentional orientation and allocation during temporal processing.

### Conclusion

It is becoming increasingly clear that the relationship between physiological arousal and emotional distortions to time is more complex than previously imagined. On the one hand, for high arousal negative stimuli, a consistent pattern is emerging in which the subjective lengthening of the perceived duration of these stimuli is predicted by the SNS reactivity they evoke. On the other hand, for low arousal negative stimuli, or high and low arousal positive stimuli, any distortion to duration appears not to be predicted by changes in physiological arousal. The findings of the current study confirm that a simple bottom-up relationship between arousal and perceived duration is inaccurate. The findings instead support the predictions of a model in which a combination of bottom-up processes (physiological arousal) and top-down processes (threat detection) produce emotional distortions time. We therefore caution against the future assumption that emotional distortions to time are *caused* by changes in physiological arousal and encourage further work to establish other factors contributing to emotional distortions to time.

## Supporting information

S1 Data Supporting data. (SAV)

## References

[1] Dormal V, Pesenti M. Processing magnitudes within the parietal cortex. Horizons in Neuroscience Research. 2012;8:107–40.

[2] Lake JI, LaBar KS, Meck WH. Emotional modulation of interval timing and time perception. Neuroscience & Biobehavioral Reviews. 2016 5 1;64:403–20. 26972824 10.1016/j.neubiorev.2016.03.003 PMC5380120

[3] Mella N, Conty L, Pouthas V. The role of physiological arousal in time perception: psychophysiological evidence from an emotion regulation paradigm. Brain and cognition. 2011 3 1;75(2):182–7. 10.1016/j.bandc.2010.11.012 21145643

[4] Brooks CI, Church MA, Fraser L. Effects of duration of eye contact on judgments of personality characteristics. The Journal of Social Psychology. 1986 2 1;126(1):71–8.

[5] Lange-Küttner C. The importance of reaction times for developmental science: What a difference milliseconds make. International Journal of Developmental Science. 2012 1 1;6(1–2):51–5.

[6] Benguigui N, Ripoll H, Broderick MP. Time-to-contact estimation of accelerated stimuli is based on first-order information. Journal of Experimental Psychology: Human Perception and Performance. 2003 12;29(6):1083 10.1037/0096-1523.29.6.1083 14640832

[7] Land MF, McLeod P. From eye movements to actions: how batsmen hit the ball. Nature neuroscience. 2000 12;3(12):1340 10.1038/81887 11100157

[8] Droit-Volet S, Meck WH. How emotions colour our perception of time. Trends in Cognitive Sciences. 2007 12 1;11(12):504–13. 10.1016/j.tics.2007.09.008 18023604

[9] Droit‐Volet S, Brunot S, Niedenthal P. BRIEF REPORT Perception of the duration of emotional events. Cognition and Emotion. 2004 9 1;18(6):849–58.

[10] Gil S, Droit-Volet S. Emotional time distortions: the fundamental role of arousal. Cognition & emotion. 2012 8 1;26(5):847–62. 22296278 10.1080/02699931.2011.625401

[11] Tipples J. Time flies when we read taboo words. Psychonomic Bulletin & Review. 2010 8 1;17(4):563–8. 20702878 10.3758/PBR.17.4.563

[12] Droit-Volet S, Fayolle SL, Gil S. Emotion and time perception: effects of film-induced mood. Frontiers in Integrative Neuroscience. 2011 8 9;5:33 10.3389/fnint.2011.00033 21886610 PMC3152725

[13] Fayolle S, Gil S, Droit-Volet S. Fear and time: fear speeds up the internal clock. Behavioural Processes. 2015 11 1;120:135–40. 10.1016/j.beproc.2015.09.014 26440426

[14] Ogden RS, Moore D, Redfern L, McGlone F. The effect of pain and the anticipation of pain on temporal perception: A role for attention and arousal. Cognition and Emotion. 2015 7 4;29(5):910–22. 10.1080/02699931.2014.954529 25203750

[15] Tipples J. Rapid temporal accumulation in spider fear: Evidence from hierarchical drift diffusion modelling. Emotion. 2015 12;15(6):742 10.1037/emo0000079 25938616

[16] Watts FN, Sharrock R. Questionnaire dimensions of spider phobia. Behaviour Research and Therapy. 1984 1 1;22(5):575–80. 6150703 10.1016/0005-7967(84)90061-5

[17] Smith SD, McIver TA, Di Nella MS, Crease ML. The effects of valence and arousal on the emotional modulation of time perception: evidence for multiple stages of processing. Emotion. 2011 12;11(6):1305 10.1037/a0026145 22142208

[18] Droit-Volet S, Bigand E, Ramos D, Bueno JL. Time flies with music whatever its emotional valence. Acta Psychologica. 2010 10 1;135(2):226–32. 10.1016/j.actpsy.2010.07.003 20674884

[19] Ogden RS, Moore D, Redfern L, McGlone F. Stroke me for longer this touch feels too short: the effect of pleasant touch on temporal perception. Consciousness and cognition. 2015 11 1;36:306–13. 10.1016/j.concog.2015.07.006 26210078

[20] Gibbon J, Church RM, Meck WH. Scalar timing in memory. Annals of the New York Academy of sciences. 1984 5 1;423(1):52–77. 6588812 10.1111/j.1749-6632.1984.tb23417.x

[21] Matell M. S., & Meck W. H. (2004). Cortico-striatal circuits and interval timing: coincidence detection of oscillatory processes. Cognitive brain research, 21(2), 139–170. Arousal pacemaker reference 10.1016/j.cogbrainres.2004.06.012 15464348

[22] Penton-Voak IS, Edwards H, Percival A, Wearden JH. Speeding up an internal clock in humans? Effects of click trains on subjective duration. Journal of Experimental Psychology: Animal Behavior Processes. 1996 7;22(3):307 8691161 10.1037//0097-7403.22.3.307

[23] Cheng RK, Tipples J, Narayanan NS, Meck WH. Clock speed as a window into dopaminergic control of emotion and time perception. Timing & Time Perception. 2016 3 10;4(1):99–122.

[24] Darvas M, Fadok JP, Palmiter RD. Requirement of dopamine signaling in the amygdala and striatum for learning and maintenance of a conditioned avoidance response. Learning & Memory. 2011 3 1;18(3):136–43. 21325435 10.1101/lm.2041211 PMC3056517

[25] Craig AD. How do you feel? Interoception: the sense of the physiological condition of the body. Nature Reviews Neuroscience. 2002 8;3(8):655 10.1038/nrn894 12154366

[26] Craig AD. Emotional moments across time: a possible neural basis for time perception in the anterior insula. Philosophical Transactions of the Royal Society of London. Series B, Biological Sciences. 2009 7;364(1525):1933–42. 10.1098/rstb.2009.0008 19487195 PMC2685814

[27] Wittmann M. The inner experience of time. Philosophical Transactions of the Royal Society of London B: Biological Sciences. 2009 7 12;364(1525):1955–67. 10.1098/rstb.2009.0003 19487197 PMC2685813

[28] Livesey AC, Wall MB, Smith AT. Time perception: manipulation of task difficulty dissociates clock functions from other cognitive demands. Neuropsychologia. 2007 1 1;45(2):321–31. 10.1016/j.neuropsychologia.2006.06.033 16934301

[29] van Hedger K, Necka EA, Barakzai AK, Norman GJ. The influence of social stress on time perception and psychophysiological reactivity. Psychophysiology. 2017 5;54(5):706–12. 10.1111/psyp.12836 28139018 PMC5529171

[30] Piovesan A, Mirams L, Poole H, Moore D, Ogden RS. The relationship between pain induced autonomic arousal and perceived duration. Emotion. 2018 in press. 10.1037/emo0000512 30265080

[31] Meck WH. Selective adjustment of the speed of internal clock and memory processes. Journal of Experimental Psychology: Animal Behavior Processes. 1983 4;9(2):171 6842136

[32] Lang PJ, Bradley MM. Emotion and the motivational brain. Biological psychology. 2010 7 1;84(3):437–50. 10.1016/j.biopsycho.2009.10.007 19879918 PMC3612949

[33] Ochsner KN, Ray RR, Hughes B, McRae K, Cooper JC, Weber J, et al Bottom-up and top-down processes in emotion generation: common and distinct neural mechanisms. Psychological science. 2009 11;20(11):1322–31. 10.1111/j.1467-9280.2009.02459.x 19883494 PMC2858766

[34] Scherer KR, Schorr A, Johnstone T, editors. Appraisal processes in emotion: Theory, methods, research Oxford University Press; 2001 5 3.

[35] Lang PJ, Bradley MM, Cuthbert BN. International affective picture system (IAPS): Technical manual and affective ratings. NIMH Center for the Study of Emotion and Attention. 1997:39–58.

[36] Sherwood A, Dolan CA, Light KC. Hemodynamics of blood pressure responses during active and passive coping. Psychophysiology. 1990 11;27(6):656–68. 2100351 10.1111/j.1469-8986.1990.tb03189.x

[37] Berntson GG, Bigger JT, Eckberg DL, Grossman P, Kaufmann PG, Malik M, et al Heart rate variability: origins, methods, and interpretive caveats. Psychophysiology. 1997 11 1;34(6):623–48. 9401419 10.1111/j.1469-8986.1997.tb02140.x

[38] Task Force of the European Society of Cardiologiy and the North American Scciety of Pacing and Electrophysiology. Heart rate variability. Standards of measurement, physiological interpretation, and clinical use. European Heart Journal. 1996 17(3): 354–381. 8737210

[39] Angrilli A, Cherubini P, Pavese A, Manfredini S. The influence of affective factors on time perception. Perception & psychophysics. 1997 1 1;59(6):972–82. 9270369 10.3758/bf03205512

[40] Azevedo TM, Volchan E, Imbiriba LA, Rodrigues EC, Oliveira JM, Oliveira LF, et al A freezing‐like posture to pictures of mutilation. Psychophysiology. 2005 5 1;42(3):255–60. 10.1111/j.1469-8986.2005.00287.x 15943678

[41] Yartz AR, Hawk LW Jr. Addressing the specificity of affective startle modulation: Fear versus disgust. Biological psychology. 2002 2 1;59(1):55–68. 11790443 10.1016/s0301-0511(01)00121-1

[42] Jones LA, Ogden RS. Vibrotactile timing: Are vibrotactile judgements of duration affected by repetitive stimulation?. The Quarterly Journal of Experimental Psychology. 2016 1 2;69(1):75–88. 10.1080/17470218.2015.1023735 25965268

[43] Richter M. Pay attention to your manipulation checks! Reward impact on cardiac reactivity is moderated by task context. Biological Psychology. 2010 84: 279–289. 10.1016/j.biopsycho.2010.02.014 20206226

[44] Kelsey RM, Guethlein W. An evaluation of the ensemble averaged impedance cardiogram. Psychophysiology. 1990 27:24–33. 2339185 10.1111/j.1469-8986.1990.tb02173.x

[45] Tarvainen MP, Ranta-aho PO, Karjalainen PA. An advanced detrending method with application to HRV analysis. IEEE Transactions in Biomedical Engineering. 2001 49: 1272–2175. 10.1109/10.979357 12066885

[46] Gu X, Hof PR, Friston KJ, Fan J. Anterior insular cortex and emotional awareness. Journal of Comparative Neurology. 2013 10;521(15):3371–88. 10.1002/cne.23368 23749500 PMC3999437

[47] Zakay D, Block RA. The role of attention in time estimation processes. In Advances in psychology 1996 1 1 (Vol. 115, pp. 143–164). North-Holland.

[48] Gil S, Rousset S, Droit-Volet S. How liked and disliked foods affect time perception. Emotion. 2009 8;9(4):457 10.1037/a0015751 19653766

[49] Coull JT. Neural correlates of attention and arousal: insights from electrophysiology, functional neuroimaging and psychopharmacology. Progress in neurobiology. 1998 7 1;55(4):343–61. 9654384 10.1016/s0301-0082(98)00011-2

[50] Portas CM, Rees G, Howseman AM, Josephs O, Turner R, Frith CD. A specific role for the thalamus in mediating the interaction of attention and arousal in humans. Journal of Neuroscience. 1998 11 1;18(21):8979–89. 9787003 10.1523/JNEUROSCI.18-21-08979.1998 PMC6793555

[51] Vuilleumier P. How brains beware: neural mechanisms of emotional attention. Trends in cognitive sciences. 2005 12 1;9(12):585–94. 10.1016/j.tics.2005.10.011 16289871

